# How Does Environmental Regulation Affect the Green Growth of China’s Citrus Industry? The Mediating Role of Technological Innovation

**DOI:** 10.3390/ijerph192013234

**Published:** 2022-10-14

**Authors:** Hepei Zhang, Zhangbao Zhong

**Affiliations:** 1 College of Economics & Management, Huazhong Agricultural University, Wuhan 430070, China; 2 Research Center for Rural Social Construction and Management, Huazhong Agricultural University, Wuhan 430070, China

**Keywords:** environmental regulation, citrus, green growth, technological innovation

## Abstract

Exploring suitable types and intensities of environmental regulations to promote technological innovation and guide industrial green growth is an essential goal for China. This paper uses the SBM super-efficiency model with the GML index to measure the level of green growth in China’s citrus industry from 2008 to 2019, and examines the impact generated by heterogeneous environmental regulations and the mediating effect of technological innovation using a panel Tobit model. The study found that: (1) From 2008 to 2019, the green growth level of the citrus industry has gradually improved, with an average annual growth rate of 2.7%, and the contribution of technical efficiency is more significant than technological progress. (2) The green growth of the citrus industry is closely related to the intensity and type of environmental regulation. The impact of market-incentive environmental regulation has an inverted U-shape, the impact of guidance-based environmental regulation is U-shaped, and the command-and-control environmental regulation has no significant effect. (3) The mediating effect suggests that guidance-based environmental regulation promotes green growth in the citrus industry by stimulating technological innovation. In contrast, market-incentive environmental regulation inhibits technological innovation and thus discourages green growth in the citrus industry. According to the study results, the government should strive to ensure the effective implementation of environmental laws and regulations, optimize the channels and amounts of investment in environmental governance, strengthen environmental protection-related media campaigns, and guide the citrus industry to break through technological bottlenecks to promote green growth.

## 1. Introduction

The long-term reliance on factor expansion has made China’s agricultural development face a series of problems, such as low green production efficiency, tightening resource constraints, and severe environmental pollution, which affects the health of the population and is detrimental to the image of the government and social stability [1,2]. The concept of “green growth” was first put forward by the United Nations Development Programme (UNDP) in 2002. This concept is widely recognized and regarded as an essential way to achieve sustainable development [3]. The Chinese government has also been supporting this idea. The “14th Five-Year Plan and 2035 Vision Outline” states that it will accelerate the change in the development model to green and promote the development of a high-quality economy and a high level of ecological environmental protection. The 2022 Central Document No. 1 emphasizes the need to strengthen the comprehensive management of agricultural non-point source pollution and promote green growth in agriculture. Subsequently, the practice and efficiency of green growth have been a top priority for governments and scholars in environmental governance [4], and promoting the green transformation of agricultural production, improving the efficiency of agricultural production, and strengthening the prevention and control of agricultural non-point surface pollution has become the key to green growth in Chinese agriculture. In this context, the Chinese government has enacted various types of environmental regulatory policies to break the cycle of “achieving economic development at the expense of environmental pollution” and achieve green growth in agriculture [5]. However, under the dual mission of economic development and environmental friendliness, the design of environmental regulation is often tricky [6]. Achieving a balance between the two to promote green economic growth has been an essential topic in the design of environmental regulations.

Since the “Action Plan for Zero Growth of Chemical Fertilizer and Pesticide Use by 2020” issued by the Ministry of Agriculture and Rural Affairs of China in 2015, the total chemical fertilizer and pesticide applications in China’s agriculture have been decreasing year by year, falling by 12.3% and 26.4%, respectively, in five years. Food crops have achieved significant green growth driven by this target, while cash crops have seen lower levels of green growth [7]. Regarding citrus, as the fruit tree with the largest planting area and the most important economic status in China [8], its total investment in chemical fertilizer and pesticide has not decreased but increased, with an increase of 12.9% and 32.6%, respectively, in the same period [9], which has become a significant hidden danger behind the realization of the zero-growth action goal. Meanwhile, the average input of chemical fertilizer and pesticide per hectare of the citrus industry has long ranked first among the major crop species in China, in which a large amount of input redundancy has not only led to a significant decrease in citrus yields and fruit quality, but also caused long-term damage to soil structure, resulting in severe negative impacts on the economy and the environment [10,11]. Obviously, the current development of China’s citrus industry is contrary to the goal of agricultural green growth, and it is difficult to explain the increasingly strict environmental regulations. So, what is the current status of green growth in China’s citrus industry? What is the impact of environmental regulation on the green growth of citrus industry? Answering these questions is relevant to achieving coordinated growth of resources, environment, and the citrus industry. Therefore, this paper measures and analyzes the level of green growth in the main citrus-producing areas of China from 2008 to 2020 and establishes an economic model to study the impact of environmental regulations on the green growth of the citrus industry, and further discusses the specific impact mechanisms.

The rest of the paper is organized as follows: Section 2 presents the literature review and research hypotheses; Section 3 presents the data and methods; Section 4 presents the results and discussion of the empirical tests; and finally, Section 5 gives the conclusions and policy recommendations.

## 2. Literature Review and Theoretical Hypothesis

### 2.1. Literature Review

Regarding the evaluation of green growth, two approaches have been developed. One way is to measure it by a single indicator, such as energy intensity or carbon intensity [12,13], which only considers pollution emissions but not the inputs or growth in the economy. The other is through green total factor productivity (GTFP), which is mainly analyzed by the data envelopment analysis (DEA) method; this is widely used by scholars in the fields of business, education, and information management for efficiency measurement in various fields [14,15,16]. This method was first proposed by Charnes, et al. [17], and can include multiple input and output variables and does not require a specific form of the production function. However, bias may be introduced due to this method’s radial and angular selection. Tone [18] constructed a standard efficiency model of SBM with slack variables on this basis, which effectively overcomes this problem but cannot be distinguished when there are multiple decision effective units [19]. For this reason, Tone [20] further proposed the SBM super-efficiency model, but this model fails to consider non-desired outputs. Later, Tone [21] optimized it again by including non-desired outputs in the SBM super-efficiency model to incorporate environmental factors well into the efficiency measure. In addition, scholars have continuously improved the existing DEA models. Afsharian, et al. [22] applied the generalized DEA model to make the original implicit assumptions of DEA, including the linear cost and benefit functions, explicit to overcome the respective input/output factor problems related to factor selection, dual-role factors, and undesirable factors. Mosbah, et al. [23] proposed a DEA model for input/output decomposition. Due to the reality of inputs/outputs and the different levels of information of managers, managing resources through (global) efficiency scores can be limiting; therefore, decomposing these into the efficiency of input/output components will be effective in improving resource management decisions. In summary, scholars have provided excellent ideas for optimizing the DEA model. The GTFP calculated by DEA is also more comprehensive and accurate than a single indicator, which is now widely used in the industry. With people’s increasing attention on the agricultural ecological environment, some scholars have also begun to apply it to the agricultural field. For example, Chen, et al. [24] used this model to calculate China’s agricultural sector’s green total factor productivity. Shi, et al. [25] used this model to calculate the utilization efficiency of agricultural water resources in the Yangtze River Basin of China.

Regarding the factors influencing green growth, studies have shown that FDI, imports, and exports influence green growth. FDI will promote industrial agglomeration, generate competitive effects and promote economic growth, and increase regional pollution [26]. Imports may lead to pollution reduction in the importing country. However, exports are likely to lead to an increase in “pollution paradise”, which is still detrimental to environmental protection overall [27]. Thus, due to the negative externality characteristics of environmental pollution, regulating the harmonization of economic and environmental development through market-oriented factors often leads to “market failure”, and environmental regulation is one of the most direct and effective measures to solve this problem [28,29]. Rubashkina, et al. [30] studied manufacturing industries in 17 European countries and concluded that government spending on reducing environmental pollution positively impacts technological innovation but is not significant for TFP growth. Wang and Shao [31], based on data from Group 20 countries, and using the environmental policy stringency index provided by the OECD to characterize formal environmental regulation, concluded that the effect of formal environmental regulation on GTFP shows a positive contribution only at high levels, while both technology and education levels, which are informal environmental regulations, have a positive effect on GTFP. In agriculture, there is a lack of comprehensive discussion on environmental supervision at present. Ma and Tan [32] chose the number of environmental protection-related policies as a proxy variable for environmental regulation and concluded that it is beneficial for increasing GTFP in agriculture, while Xu and Yin [33] chose environmental investment in agriculture as a proxy for environmental regulation and concluded that it is detrimental to GTFP in agriculture. Huang, et al. [34] further refined the industry, using the chemical fertilizer price as a proxy variable, and found an inverted U-shaped relationship between it and the wheat GTFP.

Among the mechanisms by which environmental regulation affects green growth, technological innovation is a research hotspot. Acemoglu, et al. [35] introduced endogenous and directional technological changes into the growth model with environmental constraints and concluded that appropriate environmental regulation would trigger technological innovation. Franco and Marin [36] found that environmental regulation in downstream industries is an important driver of innovation and productivity in the industry, based on data from 13 manufacturing sectors in eight European countries. Li and Shi [37] took Chinese manufacturing and new energy enterprises as the research objects, respectively, and concluded that environmental regulation would stimulate technological innovation. However, fewer articles have examined the mechanisms of technological innovation as environmental regulation in the field of agriculture.

In conclusion, the available studies help understand the relationship between environmental regulation and green growth. However, the findings vary widely and are not generalizable due to the different ways of measuring heterogeneous environmental regulations. Moreover, there are few articles related to environmental regulation and green growth in the agricultural sector, and the research on agricultural technological innovation as the mechanism of environmental regulation is also rare. As the fruit tree with the largest planting area in China, citrus has a rapid increase in yield, while efficiency and environmental problems cannot be ignored. However, the current research on green growth of the citrus industry is still relatively weak. This paper has the following marginal contributions: (1) Chemical fertilizer and pesticide losses and carbon emissions were included as non-desired outputs in measuring citrus green total factor productivity to more objectively and comprehensively reflect the green development changes in China’s citrus industry. (2) Integrating heterogeneous environmental regulation, technological innovation, and green growth into the same framework, the impact of heterogeneous environmental regulations can be reflected more comprehensively and realistically, and the mechanism of technological innovation’s role in them can be verified. (3) The citrus industry, a highly market-oriented industry, was selected as the research object to more significantly optimize the resource allocation efficiency of the agricultural market.

### 2.2. Theoretical Mechanism

Because environmental regulations are more diverse and their effects vary significantly, scholars usually discuss environmental regulations in categories, mainly command-and-control, market-incentive, and guide-participatory [38]. Command-and-control environmental regulation mainly includes environmental protection-related laws, regulations, and rules, restraining farmers’ production behavior through mandatory regulations. It is the primary way of environmental regulation in the agricultural sector in China [39]. Market-incentive environmental regulation regulates production behavior through market-based instruments such as environmental taxes and fees and environmental investments, while agricultural production in China is now free of sewage charges and environmental taxes (except for large-scale livestock and poultry farming), so environmental investments are the main application of market-incentive regulation in Chinese agriculture. There is no unified definition of informal environmental regulation regarding guidance and participation, which mainly includes environmental protection news and publicity, environmental protection-related internet search indexes, environmental protection proposals of the National People’s Congress and the Chinese People’s Political Consultative Conference, and environmental protection petitions. Under the unique production conditions in rural areas, it is difficult to objectively reflect the impact of participatory environmental regulations such as Internet search indexes and proposal petitions in the agricultural industry due to the low quality of farmers and poor information channels [40], while the guidance-based regulation represented by environmental news publicity is a more effective measure of informal environmental regulation in the agricultural sector [41]. In summary, this study classifies environmental regulations into command-and-control environmental regulations (CER), market-incentive environmental regulations (MER), and guidance-based environmental regulations (GER), and analyzes the mechanism of action of different types of environmental regulation in turn.

CER refers to mandatory constraints such as laws, regulations, rules, and regulations related to environmental protection. By formulating agricultural non-point source pollution control policies, such as chemical fertilizer and pesticide registration systems and delimiting prohibited and restricted breeding areas, local governments control various pollution sources such as chemical fertilizer and pesticide, inhibit the emission of some pollutants from the source, indirectly restrict the entry of producers below the threshold efficiency, and squeeze out inefficient incumbent producers [42], to promote environmentally friendly production. However, the public choice theory states that governments at all levels are “rational economic people” in the political market [43] and that principal–agent problems may exist between central and local governments [44]. Moreover, as local governments make GDP growth their primary objective [45], there is a competition between “incomplete implementation” and “race to the bottom” of written legislation [46,47]. Moreover, due to the concealment, dispersion, and randomness of agricultural non-point surface pollution, the enforcement and supervision of agricultural and environmental pollution are difficult. In addition, the quality of farmers and rural infrastructure construction level is low, the information asymmetry phenomenon is prominent, laws and regulations and administrative penalties to deter the effect of guidance are limited, and the effectiveness of CER may be compromised.

MER mainly refers to market-based regulatory instruments such as fiscal spending on environmental protection. For example, the government compensates farmers for green production behaviors using “promoting governance with rewards” and “substituting rewards for subsidies”, promoting the utilization of agricultural waste as a resource and the use of ecological agricultural materials, or using financial funds to build environmental protection infrastructure such as rural roads, farmland water, and production services to reduce the production cost of agriculture and increase the profit. However, due to the typical externalities of environmental regulation results, the phenomenon of “free riders” has become the norm [48]. The increase in environmental protection investment has shared the cost of environmental pollution for farmers, making them less responsible for environmental pollution and squeezing out their motivation and resources for environmentally friendly production, thus inhibiting the green growth of the citrus industry. Therefore, there may be both positive and negative effects of MER on agricultural production, and the magnitude of both effects varies with the intensity of MER, which in turn has a nonlinear effect.

GER refers to the means of enhancing public awareness of environmental protection such as environmental news and publicity. Mainly through environmental information disclosure, a more transparent information environment is built to effectively maintain citizens’ rights to environmental information, participation, and supervision, while providing the public as well as producers with more environmental value judgments and cognitive guidance [49], effectively bringing into play the informal institutional binding force of social opinion [50]. Signaling theory suggests that the media’s promotion of positive environmental messages can strengthen public awareness of environmental protection, weaken strategic interactions between subjects, align the environmental goals of producers with those of governments at all levels, and guide producers to choose more environmentally friendly production methods and restrain governments from “imperfect enforcement” [42]. The negative environmental information propaganda will damage the reputation image of the exposed producer and have a deterrent effect on other producers in power, and the social pressure brought by this will guide producers to environmental production. Initially, there will be an increase in environmental production costs, but as environmental regulations strengthen, producers become more environmentally conscious and take the initiative to optimize resource allocation and improve environmental protection, compensating for the increased costs and increasing green total factor productivity. Therefore, GER may have positive or negative effects on the aforementioned environmental regulations and vary with the intensity of GER, eventually producing a U-shaped or inverted u-shaped nonlinear effect. On this basis, Hypothesis 1 is proposed.

**Hypothesis** **1.** *There is a non-linear relationship between heterogeneous environmental regulations and citrus green growth.*

**Figure 1:**
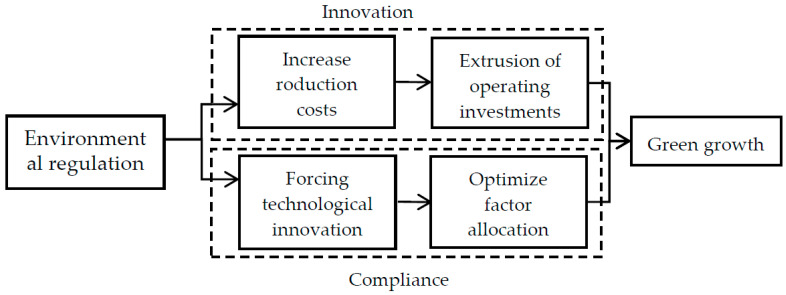
Effect path of environmental regulation on green growth.

From a static perspective, the neoclassical view is that environmental regulation has a “compliance cost” effect, leading to increased operating costs and thus crowding out R&D investment, which discourages technological innovation and reduces productivity and competitiveness, a view mainly represented by Jaffe [51] and supported by subsequent studies by many scholars [52,53]. In contrast, a series of scholars, represented by Porter [54], analyze the issue from a dynamic perspective and believe that environmental regulation with reasonable design can produce an “innovation compensation” effect. It forces producers to innovate, reduce production costs, and improve production quality [35,55]. In the agricultural field, increased environmental regulations will encourage farmers to use green farming materials or adopt green production methods, which will bring “compliance costs” to farmers, making them increase production costs or reduce business returns, squeezing out inputs for technology and management, and indirectly inhibiting green growth. However, there may also be incentives for farmers to adopt chemical fertilizer and pesticide reduction, efficiency technologies, and agricultural waste recycling technologies for profit maximization, resulting in an “innovation compensation” effect; this will optimize factor allocation and reduce pollution emissions through technological improvements, which is conducive to green growth (Figure 1). Furthermore, technological innovation promotes green growth by improving the utilization rate of agricultural factors and reducing agricultural non-point source pollution [56]. In summary, technological innovation is essential for environmental regulation to play a role. Based on this, Hypothesis 2 is formulated.

**Hypothesis** **2.** *Technology innovation plays an intermediary role in the process of environmental regulation affecting the green growth of citrus.*

## 3. Materials and Methods

### 3.1. Measurement and Analysis of Green Growth in Citrus Industry

#### 3.1.1. SBM Super-Efficiency Model including Non-Desired Outputs

The SBM super-efficiency model includes non-expected outputs proposed by Tone [21], which can be applied to both multiple-input and multiple-output models, overcoming the possible bias caused by radial and perspective, as well as taking into account environmental factors and distinguishing multiple decision effective units; this is more comprehensive and accurate than a single indicator and is now used in the measurement of agricultural GTFP. Therefore, this paper adopts the method to measure the citrus industry’s GTFP to characterize the citrus industry’s green growth. The contents of the model are as follows.

(1) $$\rho^{*}=\min\frac{\frac{1}{m}\sum_{i=1}^{m}x^{-}/x_{ik}}{\frac{1}{r_{1}+r_{2}}\left(\sum_{s=1}^{r_{1}}y^{-d}/y_{sk}^{d}+\sum_{q=1}^{r_{2}}y^{-u}/y_{qk}^{u}\right)}$$

$$s.t.\;x^{-}\geq \sum_{j=1,\neq k}^{n}x_{ij}\lambda_{j},y^{-d}\leq \sum_{j=1,\neq k}^{n}y_{sj}^{d}\lambda_{j},y^{-u}\geq \sum_{j=1,\neq k}^{n}y_{qj}^{u}\lambda_{j}$$

$$x^{-}\geq x_{k},y^{-d}\leq y_{k}^{d},y^{-u}\geq y_{k}^{u}$$

$$\lambda_{j}\geq 0,i=1,2,\cdots ,m;j=1,2,\cdots ,n,j\neq 0$$

$$s=1,2,\cdots ,r_{1};q=1,2,\cdots ,r_{2}$$

In Equation (1): $\rho^{*}$ is the target efficiency value; x, $y^{d}$ and $y^{u}$ are the input, desired output and non-desired output, respectively; $x^{-}$, $y^{-d}$ and $y^{-u}$ are the input slack, desired output slack and non-desired output slack, respectively; λ is the weight vector; the subscript “k“ in the model indicates the evaluated decision unit.

#### 3.1.2. GML Index

Equation (1) can measure the static efficiency level under the given technological conditions of each unit, but it cannot reflect the dynamic changes in inefficiency. Therefore, based on the Malmquist productivity index, Chung, et al. [57] proposed an ML index that considers resource consumption and environmental pollution. Subsequently, Oh [58] proposed the Global Malmquist–Luenberger (GML) productivity index, which takes the sum of the periods as a reference and can avoid the linear programming nonsolution and “technological regression”, and the GML index is transferable and can be multiplied cumulatively. Therefore, the GML index measures the GTFP, which is defined as Equation (2):

(2) $$GML^{t,t+1}\left(x^{t+1},y^{t+1},b^{t+1};x^{t},y^{t},b^{t}\right)=\frac{1+D_{G}^{T}\left(x^{t},y^{t},b^{t}\right)}{1+D_{G}^{T}\left(x^{t+1},y^{t+1},b^{t+1}\right)}$$

Further, the GML index can be decomposed into technical progress (GTC) and technical efficiency (GEC). All indexes take values greater than 0. A value greater than 1 indicates that the corresponding index is optimized, while the opposite indicates that degradation has occurred.

(3) $$\begin{array}{l} GML^{t,t+1}\left(x^{t+1},y^{t+1},b^{t+1};x^{t},y^{t},b^{t}\right)=\\ =\frac{1+D_{C}^{t}\left(x^{t},y^{t},b^{t}\right)}{1+D_{C}^{t+1}\left(x^{t+1},y^{t+1},b^{t+1}\right)}\times \left[\frac{\left(1+D_{G}^{T}\left(x^{t},y^{t},b^{t}\right)\right)/\left(1+D_{C}^{t}\left(x^{t},y^{t},b^{t}\right)\right)}{\left(1+D_{G}^{T}\left(x^{t+1},y^{t+1},b^{t+1}\right)\right)/\left(1+D_{C}^{t+1}\left(x^{t+1},y^{t+1},b^{t+1}\right)\right)}\right]\\ =\frac{TE^{t+1}}{TE^{t}}\times \left[\frac{BPG_{t+1}^{t,t+1}}{BPG_{t}^{t,t+1}}\right]=GEC^{t,t+1}\times GTC^{t,t+1} \end{array}$$

#### 3.1.3. Selection of Indicators

The “China Citrus Advantageous Regional Layout Plan (2008–2015)” shows that the planting range of Chinese citrus is mainly concentrated in the region south of the Yangtze River, which is divided into four major planting regions—the upper and middle reaches of Yangtze River, Jiangxi Ganzhou and Hunan Chenzhou region, southeast coastal region, and Wuling Mountain region. Considering the continuity and availability of statistical data, this research selected data from Fujian, Guangdong, Guangxi, Hubei, Hunan, Jiangxi, Zhejiang, Chongqing, and eight other provinces and cities from 2008 to 2019 as the sample. The citrus planting area and total production of the eight regions during this period accounted for more than 80% of China, so the data are representative.

The input–output variables of citrus industry are mainly: (1) land cost; including the rent per acre of flowing land and the discount rent of the self-owned camp. (2) Labor cost; including the discounted price per acre of household labor and hired labor costs. (3) Material and service costs; including direct costs such as chemical fertilizer, pesticide, drainage and irrigation, machinery, fuel and power, and indirect costs such as depreciation of fixed assets per mu. (4) The expected output variable is the total output value of citrus industry. (5) Non-desired output is the amount of nitrogen and phosphorus loss, ineffective use of pesticides, and carbon emission of agricultural production (Table 1). The data involved in the amount are deflated with 2008 as the base period. Referring to the methods of Lai, et al. [59], this study uses the “unit survey and evaluation method” according to the geographical characteristics of different production areas and uses the coefficients published in the “First China Pollution Source Census—Agricultural Source Coefficient Manual” to measure the non-point source pollution emissions of the citrus industry. Using the carbon emission calculation formula of Li, et al. [60], the carbon emission of citrus production was measured. The data were mainly obtained from the “China Agricultural Yearbook” and “National Agricultural Product Cost and Benefit Data Compilation”.

#### 3.1.4. Analysis of Citrus GTFP

**Figure 2:**
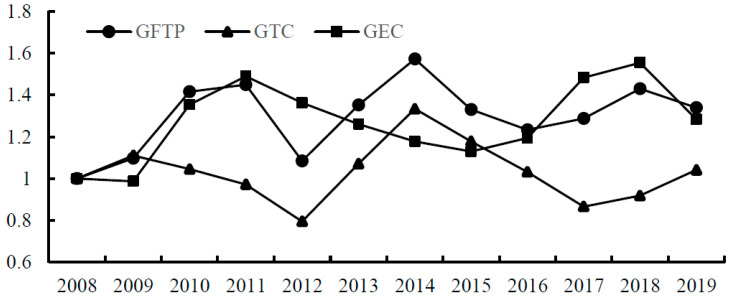
Citrus GTFP and its decomposition cumulative change.

Overall (Figure 2), the average annual growth rate of China’s citrus GTFP (GFTP) was 2.7% from 2008 to 2019. In terms of years, citrus GTFP maintained positive growth in most years and dropped significantly in 2012 and 2015. Natural disasters during the current period may be the main reason. In 2012, rare flood disasters occurred in most parts of the country. The Chongqing section of the mainstream of the Yangtze River suffered the largest flood in 30 years, and crops in Hubei, Hunan, Jiangxi, and other regions suffered much reduction. In 2015, Citrus Huanglongbing Disease spread worldwide, and the global citrus price index rose significantly. Most areas of China were severely affected, and many citrus saplings were cut down, reducing China’s citrus planting area by 7.5% in one year. Moreover, due to the long initial fruiting period of citrus, replanting costs are high, causing a long-term impact on the citrus industry. According to the decomposition of GTFP, the average annual growth rate of technological progress (GTC) and technical efficiency (GEC) is 0.4% and 2.3%, respectively. The contribution of technical efficiency is more significant than that of technological progress, which has some similarities and differences with previous studies. Early studies showed that technological progress was the primary source of citrus total factor productivity growth [61]. Still, studies based on recent data showed that technological progress gradually weakened, and technological efficiency became the main driving factor [62]. The reason may be that the marginal contribution of existing technologies gradually decreases with time. In contrast, new technologies have not achieved breakthrough progress, so farmers pay more attention to improving technical efficiency.

Based on the data from different regions, it can be concluded that citrus GTFP in Hubei, Hunan, Zhejiang, and Fujian are higher than the mean value. In contrast, the mean value of citrus GTFP in Jiangxi is lower than 1. It can be inferred that there is some coordination between citrus GTFP and the local agricultural development level and economic development level.

According to the decomposition data of regions (Table 2), Fujian, Jiangxi, Zhejiang, and Chongqing have relatively high technological progress (GTC) and relatively low technological efficiency (GEC). In the ranking of citrus production, these four provinces are the bottom four. This may be due to the small scale of the industry and the relatively low risks and costs to achieve adequate technological progress, so they pay more attention to technological progress rather than technical efficiency. This is consistent with the explanation of “small-scale technology theory”, that small-scale operation has the characteristics of labor intensity, low cost, and high flexibility and has more technical advantages than large-scale operation [63]. However, citrus production in Guangdong, Guangxi, Hubei, and Hunan ranks among the top four. The costs and risks to achieve overall technological progress are relatively high, so they focus more on improving technical efficiency than technological progress. Through the cross-validation of the two regions, it can be briefly inferred that the scale of the citrus industry in each region has a positive relationship with technical efficiency and a negative association with technological progress.

Overall, the growth in citrus GTFP was more driven by technical efficiency than technological progress. Technological progress is the core dynamic for the development of industry. The Chinese government has focused on technological innovation in the agricultural field for many years. It has established a complete agricultural R&D system from the central government to the local government, and from scientific research institutes to agricultural enterprises. The technological progress of the citrus industry has not reflected an influential role, limiting the development of the citrus industry. It has full practical significance to discuss how to promote the technological progress of the citrus industry.

### 3.2. Selection of Econometric Models

The efficiency values measured using the SBM standard efficiency model have non-negative truncation characteristics. The use of the Tobit model is more conducive to obtaining consistent and unbiased estimates. Considering the potential nonlinear relationship between the variables, a quadratic term of environmental regulations is included in the model. Based on this, a random-effects panel Tobit model was constructed as follows.

(4) $$GTFP_{it}=a_{it}+\alpha_{1}\ln ER_{j,it}+\alpha_{2}{\left(\ln ER\right)}_{j,it}^{2}+\alpha_{3}\ln X_{it}+\lambda_{it}+\pi_{it}$$

In Equation (4), $GTFP_{it}$ represents citrus GTFP, $ER_{j,it}$ is the environmental regulation variable, when *j* = 1, 2, 3, represents CER, MER and GER, respectively, $\lambda_{it}$ denotes individual error, $\pi_{it}$ denotes random error, *i* denotes province unit, *t* denotes time, $X_{it}$ denotes control variables.

Based on the “compensation for innovation” effect proposed by the Porter hypothesis, environmental regulation may act on green total factor productivity through technological innovation. Therefore, referring to the method of Wen and Ye [64], this study adopted the “three-step method” to successively construct three regression models to test the mediating effect, and the models are as follows:

(5) $$GTFP_{it}=\beta_{it}+\beta_{1}\ln ER_{j,it}+\beta_{2}\ln X_{it}+\mu_{it}+\pi_{it}$$

(6) $$\ln TI_{it}=\gamma_{it}+\gamma_{1}\ln ER_{j,it}+\gamma_{2}\ln X_{it}+\nu_{it}+\pi_{it}$$

(7) $$GTFP_{it}=\eta_{it}+\eta_{1}\ln ER_{j,it}+\eta_{2}\ln TI_{it}+\eta_{3}\ln X_{it}+\omega_{it}+\pi_{it}$$

### 3.3. Variables Definition

#### 3.3.1. Green Growth

This study used GTFP as a proxy variable for green growth. The GMI index measured in the previous section is a proxy for citrus GTFP ringgit index. Therefore, referring to Peng [65], 2008 was used as the base period and its citrus GTFP was set to 1. The concatenated multiplication method was used to find the citrus GTFP by region for each year to reflect its cumulative change.

#### 3.3.2. Environmental Regulation

Previous studies have classified environmental regulation (ER) into two dimensions: source governance, which includes environmental regulations, investment in environmental pollution control, environmental news and publicity; and end-governance, which includes revenue from sewage charges, environmental monitoring, and environmental petitions. Unlike the industrial sector, the end-of-life emissions of agricultural surface source pollution are hidden and dispersed, so the focus of environmental regulation in the agricultural industry should be on controllable source management [66]. Based on the theoretical analysis in the second part, concerning the research of Ma and Tan [32], the measure of command-and-control environmental regulation is chosen to be the number of environmental protection-related administrative regulations and environmental protection standards that belong to front-end governance; concerning the research of Xu and Yin [33], the measure of market-incentive environmental regulation is measured by the total investment in environmental governance by local governments in the current year; concerning the research of Kathuria [67], the measure of bootstrapped environmental regulation uses the number of environmental news reports in each region.

Due to data availability on environmental regulations in agriculture, the above variables are all weighted by the share of agricultural output in total local production with reference to the study by Xu and Yin [33]. Considering the dynamic nature of Porter’s hypothesis, there may be a lagged effect of environmental regulation, so this study introduces a lagged term for environmental regulation while avoiding possible endogeneity problems to a certain extent. For the possible nonlinear effects of environmental regulation, this study presents a quadratic term of environmental regulation for testing in the empirical analysis.

#### 3.3.3. Technological Innovation

The impact of environmental regulation on citrus GTFP is closely related to the output of related technical achievements in the citrus industry. In order to enhance its core competitiveness, the producers usually register patents to form technical barriers and increase profits. Therefore, the number of patents is considered a direct manifestation of technological innovation [68]. This study refers to the measure of Ma, et al. [69], using the number of citrus-related patents by province and year, as a proxy for technological innovation (TI), with data from the State Intellectual Property Office.

#### 3.3.4. Control Variables

In order to avoid errors caused by omitted variables to model estimation results, the following control variables were included in this study with reference to previous literature: (1) Average income level (wage): expressed in rural per capita disposable income. (2) Human capital (hum): expressed in terms of years of education per capita in rural areas. (3) Planting structure (str): expressed as the proportion of the citrus planting area to the planting area in the province. (4) Degree of openness (open): expressed by the ratio of each region’s total import and export value to the regional GDP after exchange rate conversion. (5) Natural factors (dis): Refer to the study of Zeng, et al. [70] to characterize the level of natural disaster rate by weighting the ratio of the affected area to the area of disaster to the total sowed crop area by 0.1 and 0.3. (6) Urbanization rate (urb): expressed as the proportion of the province’s urbanized population to the province’s total population.

### 3.4. Data Sources and Descriptive Statistics

This study selected eight provinces and municipalities in China’s prominent citrus-producing areas from 2008 to 2019 as samples. The data are mainly from the “China Agricultural Products Trade Development Report”, “China Environmental Yearbook”, “China Population and Employment Statistical Yearbook”, “China Rural Statistical Yearbook”, “China Statistical Yearbook”, and Duxiu database. To eliminate the impact of prices, the variables involved in this study are all deflated by price with 2008 as the base period. The natural results of data units and descriptive statistics are shown in Table 3. Logarithmic processing of data in empirical tests for stationarity.

## 4. Results and Discussion

### 4.1. Basic Regression Model

Table 4 shows the effects of heterogeneous environmental regulations on citrus GTFP. Models (1), (4), and (7) represent the results of the current period in terms of CER, MER, GER, and citrus GTFP, respectively. Models (2), (5), and (8) represent the results for heterogeneous environmental regulations with a one-period lag. Models (3), (6), and (9) are the results of adding a quadratic term on top of the one-period lag. The Wald test and LR test results of all models showed that the model fit was good, and the Tobit model was reasonably used.

The current term and lagged one-period term of CER are not significant, and the primary and secondary terms also fail the significance test. Becker [71] comes to the same conclusion based on data from all U.S. manufacturing plants, explaining that the effects of environmental regulation may be relatively uniform over space and time and therefore do not have a significant differential impact. The same may be true for agriculture in China due to strong policy interventions. In addition, it is also due to the difficulty of regulating agricultural surface source pollution, and regulatory constraints and penalties are ineffective. Moreover, because the citrus industry is a highly market-oriented industry, producers’ desire for profit is more prominent, and they will be more ignorant of environmental policies. In addition, due to the existence of "Incomplete implementation", the effect of environmental regulation policies is not obvious. Thresholds, crowding out low-level producers, and constraining incumbent producers have not played a significant role in the citrus industry. However, by observing its coefficients, it can be found that the coefficients are all negative, and the coefficient value of the quadratic term is greater than that of the first-order term. It shows that CER with a weak effect brings “compliance costs” but does not significantly stimulate the “innovation compensation” effect. As the intensity of regulation increases, the “innovation compensation” effect gradually compensates for the “compliance cost”, and its negative impact gradually weakens.

The current item and the lagging item of the MER are both significantly negative, indicating that producers are more responsive to the impact of environmental management investments on themselves. The coefficients of the primary and quadratic terms of MER are significantly positive and negative, indicating that the relationship between the MER and citrus GTFP shows an inverted U shape. Similar conclusions were reached by Lankoski and Thiem [72] in a study of OECD member countries, where agricultural support policies with environmental constraints were effective in increasing sustainable agricultural productivity. Research by Wang, et al. [73] on 38 OECD countries further validates this, finding that the effect of market-based environmental regulation on green GTFP changes from positive to negative beyond a certain point and that compliance costs may offset the innovation compensation effect. Therefore, with a relatively high degree of marketization and industrial income, the citrus industry is susceptible to government investment. Before the threshold, increasing investment in environmental protection can effectively reduce the degree of environmental pollution through environmental production subsidies and environmental infrastructure construction. However, with the increase in investment, citrus producers with a strong desire for profit are motivated by self-interest, and the “free rider” effect is more significant. Farmers may enjoy the effectiveness of government environmental governance while reducing their willingness to adopt green production methods, thus inhibiting the improvement of citrus GTFP.

The current period term of GER is insignificant, and the lagged period term is significantly positive, indicating that the impact of environmental news reports on citrus GTFP has a time lag effect, and its practical effect on environmental production behavior requires some time accumulation. The coefficient on the primary term of GER is significantly negative and the coefficient on the secondary term is significantly positive, showing a U-shaped relationship with citrus GTFP. In response to this result, some explanation can be provided by Campbell [74], who analyzed relevant articles from around the world. The author concluded that, in institutional contexts where monitoring of non-coercive norms is more prominent, such as by non-political organizations, institutional investors and the media, producers, and operators adopt more appropriate behaviors that reflect better environmental performance. As the income of citrus production is relatively high, citrus producers often have a heightened ability to accept information and adopt technology. Information publicity can effectively enhance environmental protection awareness and pressure on citrus producers. Although the production cost has increased, with the increase in advertising, farmers’ environmental protection awareness has increased. Due to social pressure or loss of interests, producers will be willing to improve technical efficiency or adopt green technology in time to reduce the negative impact of GER, thus enhancing GTFP of citrus.

There are results on control variables. The planting structure presents a significant adverse effect in the model. Under the current technical conditions, the citrus planting area in each province has reached relative saturation, and the area scale gain gradually disappears. Continuing to increase the proportion of citrus planting is not conducive to improving citrus GTFP. More consideration should be given to optimizing production and technology to improve the input–output and environmental protection per unit area. The openness of the model is positively significant. Smoother import and export trade can expand supply and demand, facilitate the rapid clearing of the citrus market, and improve the industry’s operational efficiency. In addition, the Chinese government strictly controls the quality of agricultural products exported, which is more conducive to the green growth. The urbanization rates were negative in the model. Rapid urbanization absorbed a large amount of rural labor and other means of production, and the substitution of labor for pesticide, chemical fertilizer, and machinery due to labor loss had a detrimental effect on citrus production and the environment.

### 4.2. Mediating Effect Test

Table 5 demonstrates the mediating effects of technological innovation. Models (10) and (11) show that the mediating effect of CER on citrus GTFP through technological innovation is not valid. Ramanathan, et al. [75] surveyed 131 manufacturing companies in the UK and also concluded that inflexible regulations such as setting standards and stipple specification standards, do not have a significant effect on the ability to innovate. Theories related to the choice of environmental regulation tools suggest that CER is highly coercive, lack incentives for developing new technologies, and have limited effects on the diffusion of existing technologies. The “innovation compensation” effect needs to meet stringent conditions. The impact of “innovation compensation” is often challenging to achieve [76]. Moreover, as the upper-level design of the central government, the legal provisions guide in terms of strategy and direction, but their effective implementation relies on local governments.

Models (12) and (13) show that MER has a significant negative impact on technological innovation, thereby inhibiting the improvement of citrus GTFP. Technological innovation played a part in the mediating role in this process, and the mediation effect accounted for 17.14% of the total effect. The research findings of Stucki, et al. [77] for Austria, Germany, and Switzerland can provide a reference, reducing the propensity to innovate if market-based environmental regulations are insufficient to induce additional demand. In addition, the “free-rider” effect of environmental pollution control investment weakens producers or stakeholders’ sense of responsibility for pollution control, reduces the demand for innovative technologies, and is not conducive to technological innovation in the citrus industry.

Models (14) and (15) show a significant positive impact of GER on technological innovation, which promotes citrus GTFP. Technological innovation played a partial mediating role in the process of environmental regulation promoting citrus GTFP growth, accounting for 13.01% of the total effect. The case study of Malaysia by Yew and Zhu [78] shows that governmental coercive actions are effective but limited for innovative environmental technologies, and non-coercive actions can fill the gaps in governmental environmental regulations. This non-coercive approach can effectively strengthen citizens’ awareness of environmental protection [79], leads producers to spontaneously improve production efficiency and environmental protection, while technological innovation, as an ideal way to improve GTFP, is an effective way for environmental regulation to play a role.

### 4.3. Robustness Analysis

The explanatory variable GTFP comprises truncated data; thus, the Tobit model chosen was only the random-effects model [80], while the panel model used a random-effects model, presupposing that the explanatory variables are related to their unobservable individual effects. After the Hausman test, the data results of this study significantly reject the original hypothesis, and the explanatory variables are related to the unobservable individual effects, so the fixed effects model should be selected for robustness testing. The results in Table 6 and Table 7 show that the degree of significance and the direction of the coefficients for both the core explanatory and control variables remained consistent, indicating that the results had no impact on the choice of a fixed-effects model or a stochastic Tobit model.

## 5. Conclusions and Policy Implications

### 5.1. Conclusions

Establishing a scientific and reasonable environmental regulation system is an effective path to green growth in Chinese agriculture, while stimulating technological innovation through environmental regulation as an essential driver of green growth is also an important issue. Therefore, this study included all three in the same analytical framework, used the SBM-GML index method to measure and analyze citrus GTFP based on data from the prominent citrus-producing areas in China, and tested the impact and mechanism of heterogeneous environmental regulations on citrus GTFP.

Based on these findings, this study draws the following conclusions. (1) The calculation and analysis show that China’s citrus industry has a good green growth trend, with technological efficiency over technological progress, in which scale efficiency tends to be saturated, and where the industrial development in each region is in line with the “small-scale technology theory”. Natural disasters, terrain characteristics, agricultural development level, and the level of economic development are potential influencing factors. (2) The benchmark regression shows that MER and GER are more effective than CER in promoting citrus GTFP. However, it is essential to note that MER has a negative effect after reaching a certain intensity. In contrast, GER needs to reach a certain intensity before it can have a positive effect. (3) The mediating effect showed that technological innovation significantly contributed to citrus GTFP. GER stimulates technological innovation, while MER conversely inhibits it, and CER has no effect on technological innovation.

### 5.2. Policy Implications

Based on these findings, this study draws the following policy Implications. (1) Improve the environmental laws and regulations to apply them in the agricultural sector effectively. Due to the difference between the central and local interests, the “inadequate implementation” of environmental laws and regulations is serious, and the effect on agriculture is lost. Especially in the citrus industry, as a highly market-oriented industry, the profit-seeking behavior of producers is more apparent, and the environmental protection laws and regulations are even more ignored, further hindering the influential role of environmental protection laws and regulations in the citrus field. Therefore, more attention should be paid to the environmental laws and regulations in the citrus field; the binding force and punishment should be strengthened, the weakness of producers’ pursuit of interests should be grasped more deeply, and more scientific and strict regulations should be formulated. In addition, the top-level design of policies and grass-roots implementation should be optimized, and the transformation of environmental laws and regulations from theoretical content to effective practice should be promoted. (2) Optimize the channels and amount of investment in environmental governance to play a positive role in environmental protection investment. Environmental protection in the field of agriculture costs a large amount money. However, due to the difference between agricultural and industrial production entities, environmental management in the agricultural sector cannot rely on government funds as in the industrial sector. Reasonable investment methods and intensity are the key “catalysts” for agricultural green production under market economy conditions. Moreover, the citrus industry is highly market-oriented and has a high industrial income. It is susceptible to government investment. Therefore, it is necessary to objectively and comprehensively consider the possible positive or negative impact of government investment in environmental protection and make targeted optimization. (3) Strengthen the media publicity and the beneficial impact on the environmental awareness of farmers. Due to the higher income of citrus production, producers generally have a higher threshold to accept all kinds of information. Environmental protection news publicity can produce a relatively improved effect. Therefore, the government should continue to strengthen environmental protection publicity, improve the breadth and depth of environmental information disclosure, fully utilize the monitoring mechanism and educational guidance of the media, and further strengthen the positive impact of environmental protection publicity on the environment. (4) Strengthen scientific and technological innovation and promote green development. Government departments should actively guide the technological innovation of the citrus industry and strengthen the guiding role of environmental regulations on technological innovation. Key citrus producing fields are encouraged to carry out technological transformation according to their endowments, increase the output of technological achievements, and carry out technological research and development based on practical problems, such as the common “Citrus Huanglongbing”, “decline disease”, and drug applications by uncrewed aerial vehicles. The aim should be to improve the conversion rate of technological achievements and fully reflect the positive role of technological innovation in citrus GTFP.

In addition, there are possibilities for further optimization in this study. This study classifies environmental regulation into three categories. The subsequent indicator system can be expanded to select more types or subdivided targeted indicators in industrial research to obtain more guiding research conclusions. In terms of data, this study chose data at the provincial level, which is not refined enough. If we can select data from companies or more refined regions, we may be able to improve the persuasive power of this article’s research., which is the next important point to focus on and overcome.

## Figures and Tables

**Table 1:** Calculation index of citrus GTFP.

Variable Name	Unit	Mean	S.D.	Min	Max
Material and Service Costs	Yuan/acre	900.17	440.83	278	1830
Labor Costs	Yuan/acre	1109.19	671.30	344	4173
Land Costs	Yuan/acre	156.05	72.09	47	340
Total output value	Yuan/acre	3068.97	1466.63	715	7539
Surface Source Pollution	kg/acre	11.75	5.57	3.01	30.66
Carbon Emissions	kg/acre	61.19	19.48	20.55	93.64

**Table 2:** Citrus GTFP and its decomposition change by Region.

	Mean	Fujian	Guangdong	Guangxi	Hubei	Hunan	Jiangxi	Zhejiang	Chongqing
GTFP	1.027	1.032	1.001	1.017	1.069	1.073	0.942	1.067	1.020
GTC	1.004	1.036	0.996	0.973	0.949	0.985	1.028	1.052	1.017
GEC	1.023	0.996	1.005	1.045	1.127	1.089	0.917	1.014	1.004

**Table 3:** Variable definition and descriptive statistics.

Variables	Mean	S.D.	Min	Max	Description
GTFP	1.392	0.546	0.487	3.009	Calculated by SBM-GML index method
CER	0.286	0.250	0.000	1.591	Local regulations, administrative regulations, and environmental protection standards
MER	13.98	6.888	4.275	36.29	The total investment in environmental governance by local governments in the current year
GER	2 047	1 061	488	5 368	Number of environmental news reported by region
wage	9 496	4 185	3 690	24 587	Rural per capita disposable income
hum	7.676	0.338	6.833	8.312	Years of education per capita in rural areas
str	1.951	1.014	0.554	5.484	Citrus planted area/total planted area
open	3.329	2.997	0.531	12.928	The total value of imports and exports/regional product
dis	4.157	3.188	0.466	17.329	(Disaster-affected area × 0.1 + Disaster-formed area × 0.3)/total sown area
urb	0.561	0.881	0.382	0.714	Urbanized Population/Total Population

**Table 4:** Impact of environmental regulation on citrus GTFP.

	*CER*			*MER*			*GER*		
	(1)	(2)	(3)	(4)	(5)	(6)	(7)	(8)	(9)
ER	−0.109			−0.562 ***			0.173		
	(0.068)			(0.122)			(0.195)		
L.ER		−0.069	−0.068		−0.399 ***	1.477 **		0.737 ***	−12.172 **
		(0.066)	(0.066)		(0.126)	(0.708)		(0.181)	(5.441)
L.ER2			−0.000			−0.386 ***			0.914 **
			(0.001)			(0.144)			(0.386)
wage	−0.093	0.302	0.295	−0.831	0.100	−0.158	−0.131	−0.044	0.117
	(0.629)	(0.732)	(0.734)	(0.719)	(0.705)	(0.690)	(0.633)	(0.755)	(0.840)
hum	2.326	0.142	0.130	2.325	0.446	1.609	1.386	0.374	1.459
	(2.175)	(2.269)	(2.270)	(1.996)	(2.143)	(2.097)	(2.186)	(2.103)	(2.149)
str	−0.303 **	−0.346 **	−0.347 **	−0.231 *	−0.248 *	−0.215 *	−0.249 *	−0.286 **	−0.198
	(0.154)	(0.140)	(0.140)	(0.125)	(0.134)	(0.129)	(0.135)	(0.124)	(0.125)
open	0.243	0.591 **	0.594 **	0.206	0.441 *	0.406 *	0.268	0.565 ***	0.559 ***
	(0.223)	(0.236)	(0.237)	(0.208)	(0.232)	(0.224)	(0.218)	(0.209)	(0.190)
dis	0.039	0.096	0.096	0.031	0.057	0.093	0.080	0.025	0.033
	(0.089)	(0.086)	(0.086)	(0.078)	(0.081)	(0.079)	(0.086)	(0.079)	(0.073)
urb	−1.652	−4.171 **	−4.195 **	−0.251	−3.525 **	−2.990 **	−1.740	−4.673 ***	−4.838 ***
	(1.377)	(1.686)	(1.695)	(1.425)	(1.578)	(1.507)	(1.362)	(1.548)	(1.587)
_cons	−4.518	−3.542	−3.467	4.334	−1.324	−3.088	−3.104	−6.801	35.167 *
	(7.270)	(7.691)	(7.706)	(7.378)	(7.385)	(7.191)	(7.001)	(7.466)	(19.062)
Time-fixed effect	YES	YES	YES	YES	YES	YES	YES	YES	YES
Wald	37.443 ***	33.820 ***	33.846 **	64.742 ***	46.359 ***	58.093 ***	33.492 ***	57.693 ***	77.519 ***
LR	17.101 ***	23.623 ***	23.335 ***	23.741 ***	24.414 ***	27.534 ***	17.803 ***	39.674 ***	32.433 ***

*, **, and *** indicate statistical significance at the 10%, 5%, and 1% levels, respectively. Standard errors are in parentheses.

**Table 5:** Intermediary effect of technological innovation.

	*CER*		*MER*		*GER*	
	*TI*	*GTFP*	*TI*	*GTFP*	*TI*	*GTFP*
	(10)	(11)	(12)	(13)	(14)	(15)
L.ER	−0.091	−0.029	−0.324 **	−0.291 **	0.761 ***	0.562 ***
	(0.067)	(0.060)	(0.136)	(0.121)	(0.211)	(0.180)
TI		0.436 ***		0.346 ***		0.309 ***
		(0.098)		(0.093)		(0.092)
wage	0.335	0.067	0.175	−0.054	−0.068	−0.180
	(0.583)	(0.651)	(0.597)	(0.640)	(0.432)	(0.712)
hum	−2.467	1.683	−2.763	1.730	−3.943 **	1.588
	(2.241)	(2.044)	(2.181)	(1.998)	(1.967)	(2.011)
str	−0.237 *	−0.213 *	−0.176	−0.163	−0.164	−0.204 *
	(0.140)	(0.128)	(0.139)	(0.126)	(0.117)	(0.119)
open	0.213	0.442 **	0.159	0.332	−0.047	0.457 **
	(0.199)	(0.215)	(0.203)	(0.218)	(0.194)	(0.199)
dis	0.048	0.078	0.041	0.044	−0.018	0.022
	(0.087)	(0.078)	(0.089)	(0.076)	(0.094)	(0.074)
urb	−2.043	−2.711 *	−1.937	−2.452 *	−1.057	−3.390 **
	(1.629)	(1.475)	(1.536)	(1.447)	(1.243)	(1.485)
_cons	4.141	−5.196	7.012	−3.414	4.864	−7.075
	(6.811)	(6.906)	(7.012)	(6.831)	(6.079)	(7.024)
Time-fixed effect	YES	YES	YES	YES	YES	YES
Wald	49.683 ***	63.135 ***	50.840 ***	67.895 ***	57.125 ***	77.245 ***
LR	22.332 ***	33.250 ***	27.439 ***	29.531 ***	1.707 *	40.919 ***

*, **, and *** indicate statistical significance at the 10%, 5%, and 1% levels, respectively. Standard errors are in parentheses.

**Table 6:** Impact of environmental regulation on citrus GTFP (Fixed effect model).

	*CER*			*MER*			*GER*		
	(16)	(17)	(18)	(19)	(20)	(21)	(22)	(23)	(24)
ER	−0.104			−0.637 ***			0.252		
	(0.076)			(0.136)			(0.222)		
L.ER		−0.058	−0.058		−0.390 ***	1.471 *		0.782 ***	−18.621 ***
		(0.074)	(0.075)		(0.145)	(0.813)		(0.208)	(5.643)
L.ER2			−0.000			−0.384 **			1.374 ***
			(0.001)			(0.165)			(0.399)
Control variables	YES	YES	YES	YES	YES	YES	YES	YES	YES
R^2^ (within)	0.367	0.364	0.364	0.479	0.414	0.461	0.329	0.466	0.551

*, **, and *** indicate statistical significance at the 10%, 5%, and 1% levels, respectively. Standard errors are in parentheses.

**Table 7:** Intermediary effect of technological innovation (Fixed effect model).

	CER		MER		GER	
	*TI*	*GTFP*	*TI*	*GTFP*	*TI*	*GTFP*
	(25)	(26)	(27)	(28)	(29)	(30)
L.ER	−0.097	−0.017	−0.300 *	−0.292 **	0.514 **	0.630 ***
	(0.075)	(0.068)	(0.159)	(0.140)	(0.238)	(0.204)
TI		0.421 ***		0.328 ***		0.296 ***
		(0.114)		(0.108)		(0.105)
Control variables	YES	YES	YES	YES	YES	YES
R^2^ (within)	0.441	0.481	0.428	0.490	0.438	0.527

*, **, and *** indicate statistical significance at the 10%, 5%, and 1% levels, respectively. Standard errors are in parentheses.

## Data Availability

Not applicable.
